# A Review on Novel Techniques Used for Drying Medicinal Plants and Its Applications

**DOI:** 10.1155/ijbm/4533070

**Published:** 2025-07-09

**Authors:** Ansu Sara Eapen, Yuvraj Khasherao Bhosale, Swarup Roy

**Affiliations:** ^1^Department of Food Technology and Nutrition, School of Agriculture, Lovely Professional University, Phagwara 144411, Punjab, India; ^2^Agricultural and Food Engineering Department, Indian Institute of Technology Kharagpur, Kharagpur 721302, West Bengal, India

**Keywords:** bioactive compound, medicinal plants, novel drying, preservation

## Abstract

The drying of medicinal plants is a crucial step in their processing since it preserves the active ingredients and increases their shelf life. Traditional drying methods often exhibit limitations such as extended drying time, loss of bioactive components, and decreased product quality. Novel drying methods have recently come to light as possible alternatives for drying medicinal plants. Reduced drying time, greater bioactive ingredient preservation, and improved product quality are just a few benefits of these novel methods. The bioactive components of medicinal plants can be preserved using these modern drying methods, which also provide possibilities for improved processing efficiency, less energy utilization, and increased product stability. However, while choosing a drying method, it is important to take into account the distinctive features of the medicinal plant, the desired quality attributes, and the economic feasibility. This review gives an overview of novel techniques such as microwave drying, vacuum drying, freeze drying, refractance window drying, Osmo drying, supercritical CO_2_ drying, and spray drying for drying medicinal plants and some potential applications.

## 1. Introduction

A plant species that has been traditionally utilized or evaluated for its therapeutic qualities is referred to as a medicinal plant. These plants possess bioactive compounds that can be utilized to cure or prevent illnesses, reduce symptoms, or enhance general well-being. In local communities all around the world, medicinal plants have long been used as a source of healing. The vast majority of people still use it as their primary healthcare method, and it is still important today as a source for the development of new drugs [1]. Plant-based remedies are vital to all therapeutic options, particularly in rural regions, due to their accessibility, affordability, and lack of adverse effects. Furthermore, a variety of plants offer a rich source of bioactive compounds that have potent pharmacological effects without any negative side effects. This is very important as nowadays people are more health conscious about the side effects of modern allopathy medicines.

The therapeutic ability of medicinal plants is due to their bioactive compounds such as alkaloids, flavonoids, tannins, and polyphenols [2]. Willow bark, turmeric, and ginger are a few plants that have natural analgesic characteristics that can help with pain relief. Some herbs and plants, like elderberry, garlic, and echinacea, naturally strengthen the immune system [3]. Inflammation can be reduced with the aid of plants with natural anti-inflammatory qualities, such as chamomile, ginger, and turmeric. To maintain digestive health and ease symptoms of digestive problems, many medicinal herbs can be employed. Aloe vera, fennel, and peppermint are some examples. Lavender, chamomile, and passionflower are a few examples of medicinal plants that might help lessen the effects of stress and anxiety. Aloe vera for burns and comfrey for wounds are two examples of plants with qualities that might help soothe and treat skin issues. Some medicinal plants, including green tea, gooseberries, turmeric, and others, have antioxidant properties [4, 5].

Problems associated with green medicinal plants is that there is the probability for the presence of foodborne pathogens, changes due to enzyme activity, and another problem being the rapid quality deterioration that occurs soon after harvest, that is after harvesting, the plant will dehydrate, wilt, degrade, and lose some nutrients. To solve this, it is essential to reduce the moisture content. Water activity is a significant component in the biological material which affects the shelf life and physical and chemical properties of medicinal plants. Hence, drying is done to remove water to prevent enzymatic and microbial activity which consequently leads to preserving the quality and extending the shelf life. Extended drying can have some negative impacts, including degradation of phytochemicals, loss of nutritional and sensory quality, increased shrinkage, formation of unfavorable compounds, decreased rehydration ratio, etc. [6].

There have been several drying methods that seek to increase drying efficiency while also enhancing product quality [4, 7]. In order to quickly reduce the moisture content without compromising the quality of the active ingredients in the medicinal plant, appropriate dryers are required. These dryers should use the most suitable temperature, velocity, and humidity values [8]. This will also efficiently reduce the weight and volume of the plant, which has favorable effects on transport and storage; it may also help ensure a steady supply and ease the marketing of plants. Different drying techniques are available, and each has its own characteristics. Due to the low vacuum level, vacuum drying is ideal for materials that readily react with oxygen. However, due to the lack of air and the difficulty of heat convection, vacuum drying requires a prolonged drying period [9]. While hot-air drying was more effective at preserving flavonoids, freeze drying was appropriate for retaining polyphenolic compounds. In addition to these, nowadays other novel methods like microwave drying, fluidized bed drying, spray drying, refractance window (RW), etc., which are superior compared to the conventional methods, are used. To overcome the limitations of one method, drying techniques can be used in combinations also. For example, microwave vacuum drying is used for galangal (*Alpinia galanga* L.) [10].

Thus, it is important to note that proper postharvest operations, particularly appropriate drying methods, can have a significant impact on the quality and safety of medicinal plants. The present review aims to determine novel drying techniques used for various medicinal plants and its applications. Drying medicinal plants is a practice that has been in use for centuries. In this review, the latest techniques for drying medicinal plants, including RW drying, microwave drying, and hybrid drying, are discussed. The impact of these techniques on the quality of the dried product and the preservation of its medicinal properties from different studies are included. Various findings suggest that these novel techniques have their applications, and offer a promising alternative to traditional drying methods, paving the way for a more sustainable and efficient approach to medicinal plant drying. This paper presents new technologies for drying medicinal plants, addressing the critical need for effective preservation methods in plant-based products. Traditional drying methods often compromise the bioactive compounds essential for therapeutic efficacy. The paper explores modern approaches, such as microwave drying, vacuum drying, freeze drying, RW drying, osmo drying, supercritical CO_2_ drying, spray drying, and hybrid drying techniques, which improve the retention of phytochemicals while ensuring optimal removal of moisture. The review outlines several key sections, including an overview of medicinal plants and their bioactive compounds, followed by a brief history of conventional drying techniques to contextualize the development of preservation methods. The main focus of the paper is on novel drying technologies, and each method is analyzed in terms of its mechanism, applications, advantages, and limitations. If we can effectively preserve the active components in medicinal plants, we can similarly preserve the temperature-sensitive food products.

## 2. Medicinal Plants

Medicinal plants are those that have long been utilized for their healing qualities in diverse cultures. These plants have chemicals called bioactive compounds that interact with the body to cause physiological effects. Medicinal plants have long been acknowledged as valuable tools for maintaining and enhancing health in traditional medical systems [11]. Throughout ancient medical systems like Ayurveda, traditional Chinese medicine, and native American medicine, the utilization of medicinal plants has been a fundamental component for thousands of years [12]. Unlike synthetic drugs, which are often isolated compounds, medicinal plants contain a complex mixture of bioactive compounds that work synergistically to produce a therapeutic effect [13]. According to these systems, disease prevention and treatment revolve around restoring the harmony between the body, mind, and spirit. Medicinal plants play a crucial role in these systems by providing a natural and holistic approach to healing. They are considered as gifts from nature since they have a variety of therapeutic qualities that assist in the innate healing ability of our body [14]. They are believed to work in association with the body, addressing the root cause of the disease rather than just relieving the symptoms. Apart from their therapeutic qualities, medicinal plants are often more accessible and affordable in comparison with modern pharmaceutical medications.

The therapeutic effects of medicinal plants are due to their bioactive components. These compounds interact with specific receptors in the body, modulating biochemical pathways and physiological processes to restore health. Medicinal plants contain thousands of bioactive chemicals, each with specific properties and possible health benefits. The most common classes of bioactive compounds found in medicinal plants include alkaloids, terpenes and terpenoids, phenolic compounds, etc. [15]. Some common medicinal plants, their bioactive compounds, and important pharmacological effects are given in Table 1. Alkaloids are substances that contain nitrogen and frequently have significant physiological effects on the body. They are found in many medicinal plants, such as opium poppy (*Papaver somniferum*), which contains morphine and codeine, and cinchona (*Cinchona* spp.), which contains quinine [52]. Flavonoids are a diverse group of compounds that are widely distributed in nature. They are present in plants like citrus fruits, green tea, and *Ginkgo biloba* and are well known for their anti-inflammatory and antioxidant properties [53]. Terpenes are hydrocarbons that give plants their distinct aromas and flavors. They are present in plants like lavender (*Lavandula angustifolia*), rosemary (*Rosmarinus officinalis*), and cannabis (*Cannabis sativa*) and have a wide range of therapeutic properties [54]. Phenolic compounds are aromatic compounds that have antioxidant and anti-inflammatory effects. They are found in many medicinal plants such as syringic acid and gallic acid from *Moringa oleifera*, rosmarinic acid from *Rosmarinus officinalis* L. and *Mentha canadensis* L., and vanillin from *Thymus vulgaris* [55].

Bioactive compounds have a wide range of therapeutic activities, including anti-inflammatory, antioxidant, antidiabetic, antimicrobial, anticancer, and immunity-enhancing properties [56]. Chronic inflammation is the basis of numerous health issues, ranging from cardiovascular diseases to autoimmune disorders [57]. It has been found that the bioactive substances in medicinal plants have exceptional anti-inflammatory properties. For example, curcumin, a bioactive compound present in turmeric, has been extensively studied for its anti-inflammatory effects. It inhibits the production of inflammatory molecules in the body, reducing inflammation and reducing associated symptoms [58]. Chamomile (*Matricaria recutita*), sometimes referred to as chamaemelon, is an important plant that is well known for its antibacterial and anti-inflammatory properties, which help it to effectively heal burns, ulcers, and wounds [59]. Other bioactive substances with strong anti-inflammatory qualities include quercetin in onions and resveratrol in grapes [60]. Oxidative stress and cell damage can be caused by free radicals, which are unstable molecules generated in our body as a result of natural processes or external influences like pollution and UV radiation [61]. Several diseases, such as cancer, heart problems, and neurological issues, are linked to this oxidative stress.

Bioactive compounds act as powerful antioxidants, neutralizing free radicals and preventing cellular damage. For example, green tea contains the bioactive component epigallocatechin gallate (EGCG), which is well known for its antioxidant properties [62]. Regular consumption of green tea rich in EGCG can help protect against oxidative stress and improve overall health. Bioactive compounds derived from medicinal plants have shown potential in managing diabetes by regulating blood sugar levels [63]. For instance, berberine, a bioactive substance present in *Tinospora cordifolia* and *Berberis aristata* among other plants, has been shown to have antidiabetic potential [64]. It lowers blood sugar levels, increases cell uptake of glucose, and helps increase insulin sensitivity. The rise of antibiotic resistance has fueled the search for alternative antimicrobial agents. Bioactive compounds have emerged as promising options due to their ability to inhibit the growth of harmful bacteria, fungi, and viruses. Certain bioactive substances work by rupturing the cell walls of pathogens, while some others interfere with their metabolic functions [65]. For example, allicin in garlic exhibits potent antimicrobial activity against a wide range of microorganisms [66]. Additionally, tea tree oil contains terpinen-4-ol that possesses antiviral, antibacterial, and antifungal properties [67]. Cancer is a complicated condition characterized by uncontrolled cell growth and the ability to invade surrounding tissues. By focusing on several pathways involved in tumor growth and progression, bioactive compounds have shown possibilities in the prevention and treatment of cancer. For example, curcumin, present in turmeric and paclitaxel, a terpenoid found in Pacific yew (*Taxus brevifolia*), has exhibited anticancer properties by inhibiting the growth of cancer cells, inducing apoptosis, and reducing tumor angiogenesis [68]. Other than these, medicinal plants also offer antipyretic, antiallergic, neuroprotective, immunomodulatory, and other properties. Apart from their therapeutic qualities, medicinal plants are often more accessible and affordable in comparison with modern pharmaceutical medications.

## 3. A Brief History of Drying Methods

The history and evolution of drying methods for plants show human efforts to preserve food and therapeutic herbs [69]. It began with ancient civilizations utilizing the natural heat of the sun. As early as 20,000 BC, humans began the practice of sun-drying meat, a technique that laid the foundation for future preservation methods. By 12,000 BC, communities in the Middle East and Europe were utilizing sunlight to dry their agricultural produce [70]. The Egyptians and Greeks documented early sun-drying methods, spreading plant materials on flat surfaces to dehydrate them naturally. Monks hung herbs in well-ventilated attics, preserving their medicinal and culinary properties throughout harsh winters [71]. The 19th century marked a pivotal era in the advancement of drying techniques. In 1800, constructing a brick dryer represented one of the earliest mechanical attempts to improve grain drying efficiency. Following this in 1856, Gail Borden Jr. made significant advancements with his meat biscuit, which is an example of an early effort to develop a vacuum-based water-removal process [71]. The Industrial Revolution marked a significant shift with the introduction of mechanized drying. Kilns and ovens provided faster, more efficient drying methods, which are crucial for industries like tobacco and hops [72]. These advancements laid the groundwork for modern dehydration techniques, which emerged prominently in the 20th century [73]. Over 85% of industrial dryers are of the convective type, utilizing hot air or combustion gases for heat transfer. The drying process, which involves simultaneous heat and mass transfer, is energy-intensive, accounting for about 12%–20% of energy usage in the manufacturing sector. Maintaining product quality by preserving nutritional, functional, and sensory attributes is a significant concern [73]. In response, recent technological advancements have emerged in food drying, focusing on enhanced pretreatments, techniques, and equipment. These innovations aim to improve drying efficiency while preserving product integrity, addressing the industry's pressing need for more effective and energy-efficient drying solutions [70].

## 4. Novel Drying Techniques

Appropriate preservation of these medicinal plants while maintaining their bioactive constituents is essential in achieving the full potential of these plants. Traditional drying techniques like solar or hot-air-oven drying are still extensively employed; however, prolonged exposure to heat and light frequently causes the loss of volatile bioactive components, their oxidation, and a reduction in effectiveness. Novel drying methods that offer more effective and controlled drying for medicinal plants have emerged to overcome these challenges [74]. These advanced techniques are designed to preserve maximum amount of bioactive compounds as possible while minimizing their degradation or change throughout the drying process. Novel drying techniques, its suitable drying conditions, effects on bioactive compounds, and some medicinal plants to which it is applied are given in Table 2.

### 4.1. Microwave Drying

**Figure 1:**
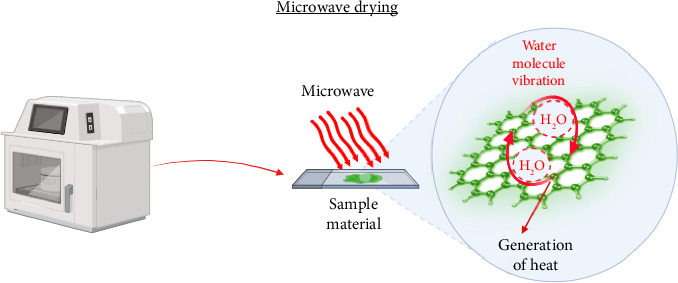
Schematic representation of the microwave drying process.

During microwave drying, electromagnetic energy is converted to thermal energy [98]. Electromagnetic waves having a frequency range of roughly 0.3–300 GHz are referred to as microwaves [99]; 0.915 GHz and 2.45 GHz are the permitted and utilized frequencies for industrial applications [100]. In microwave drying, the energy is primarily absorbed by the moist plant material that is kept in the chamber since the microwave-emitted radiation is restricted within the cavity, and there is little heat loss by means of conduction or convection. Additionally, this energy is mostly absorbed by water in the material, raising temperature, evaporating some water, and lowering moisture content. Many food molecules, principally water and to a limited extent fat and sugars are electric dipoles. They revolve in an effort to align themselves with the alternating electric field produced by the microwave beam while absorbing energy from it [101]. These rotating molecules hit and cause the movement of additional molecules, generating heat through friction. The mechanism of microwave drying is explained in Figure 1. The efficiency of microwave drying depends on equipment structure, drying conditions (microwave power, frequency, temperature, and air velocity), material properties, pretreatments given to the material, etc. [102].

In comparison to traditional sun drying and hot-air drying, microwave-dried products had less shrinkage, a shorter drying time, better color retention, and a higher rehydration capacity [79]. In a study on the essential oil composition of basil (*Ocimum basilicum* L.) after microwave drying using 1150 W for 30 min, a higher concentration of terpinolene, thymol *α*-*trans*-bergamotene, *cis*-Muurola-4(14),5-diene, 1-epi-cubenol was obtained [100]. In a different study on *Lavandula angustifolia*, 38 components were identified and quantified, accounting for 98.2% of the total oil, after microwave drying for 5 min at 55% energy of 900 W. Additionally, when temperature and microwave power increased, the essential oil content decreased [103]. A research work on *Hovenia dulcis* microwave drying at 600 W exhibited higher phenolic content, antioxidant, and antiglycation activities [104]. In a study of drying Shatavari (*Asparagus racemosus* wild) root using microwave powers of 1000 and 1500 W, the total phenolic content (TPC) and total flavonoid content (TFC) were highest in the 1000 W drying [105]. In a comparative study on the effects of the drying method on color, morphological characteristics, bioactive composition, and aroma of saffron, microwave drying time is quite short, retaining the greatest amount of total crocins and the greatest amount of aroma compounds [80].

#### 4.1.1. Applications

Considering industrial applications, microwave drying is still a new technology and requires electric power, which makes it expensive compared to traditional drying methods. This method is commonly used for herbal medicines as it enables a quick and efficient drying process. Microwave drying offers several advantages, such as reduced drying time, maintenance of the color of plant material, and preservation of its bioactive compounds due to minimal exposure to high temperatures. Additionally, microwave drying reduces microbial activity and effectively removes unwanted moisture, making it suitable for the preservation of medicinal plants. It has been discovered to be a productive and successful way to dry medicinal plants like basil, chamomile, mint, sage, etc. It is a thermal technology, and because of that, there are still chances for thermal degradation of temperature-sensitive compounds. Oxidation of some phytochemicals can also occur due to the presence of air [102]. To improve drying performance and product quality, microwave drying can be combined with other drying methods such as tray drying, vacuum drying, hot-air drying, etc.

### 4.2. Vacuum Drying

**Figure 2:**
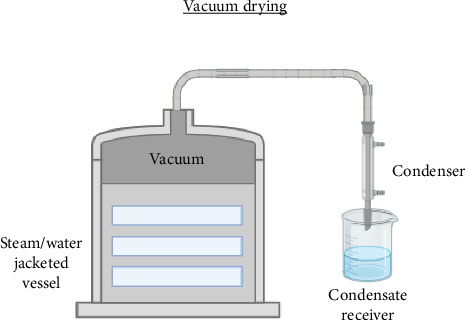
Schematic representation of the vacuum drying process.

The amount of oxygen that contacts the substance being dried during vacuum drying is limited. Reduced pressure causes moisture in the product to evaporate at a lower temperature, improving product quality [80]. The method of vacuum drying is given in Figure 2. It may be vital to avoid using too much heat when drying powder for several reasons, from product quality to safety. By lowering the moisture content of the plant materials without considerably lowering (oxidizing) the concentration of active medicinal components in the dried plant materials, vacuum drying helps in maintaining the medicinal properties of the plant [106].

A superior antioxidant profile from Lamiaceae herbs was shown by vacuum drying, which also found to exhibit very good nutrient retention in certain commonly consumed herbs such as basil, drumstick, and mint leaves [107]. After vacuum drying, the color of lemon balm and sweet basil leaves did not find to change significantly, indicating that the pigments were not much degraded. Additionally, in the same study, vacuum-dried peppermint was found to contain the most rich extract that had the highest polyphenol and flavonoid content [78]. In a similar study regarding the vacuum drying of lemongrass, a final moisture content of 8%–10% was obtained without much loss in color due to enzymatic browning [76]. In another study on the physical and phytochemical properties of *Clinacanthus nutans*, the vacuum drying method gave good retention of color and a high rehydration ratio, and the dried leaves samples gave better TPC and antioxidant activity than fresh leaves [79]. The vacuum drying method was found to retain the original color, appearance, and desirable aroma in *Anoectochilus roxburghii* plants but most importantly, the vacuum drying method maintains the abundance of active ingredients (polysaccharide, flavonoid, and phenolic compounds) [81].

Through faster moisture migration from the center to the exterior, increase in both temperature and vacuum throughout the vacuum drying process allowed for a shorter drying period [108]. Aside from improving the development of the Maillard reaction/caramelization products, drying at high temperatures also causes the breakdown of complex polyphenols into low-molecular-weight antioxidant activity–containing molecules, which will exhibit a slight rise in antioxidant activity as the temperature rises [109]. This, however, will cause temperature-sensitive substances, such as ascorbic acid, chlorogenic acid, flavanols, and phenolic acids, to degrade. Hence, it is better to use lower temperature by increasing vacuum, and at the same time reduced time to preserve these phytochemicals [110]. Since vacuum is provided, loss of ascorbic acid and other oxygen-sensitive compounds due to oxidation is reduced [111]. The degradation of ascorbic acid in medicinal plants during drying is primarily influenced by various factors such as temperature, metal ions, and oxygen exposure. Ascorbic acid can be converted into dehydroascorbic acid in the presence of oxygen and at higher temperatures [112]. Dehydroascorbic acid is an unstable form of ascorbic acid and further degrades into various degradation products, including diketogulonic acid and oxalic acid, which are not nutritionally significant. This is prevented in the case of vacuum drying. Higher temperature was found to result in loss of volatile oil content since oil cells break from heat, which releases volatile oil [113]. They are of great importance in the case of medicinal plants. Lower temperatures were found to have a superior influence on the color, appearance, and texture of vacuum-dried leaves compared to higher temperatures. This was related to vacuum drying conditions, in which the wet material is dried at subatmospheric pressures, preventing color deterioration and resulting in superior quality [108]. Less exposure to Maillard and nonenzymatic browning reactions during drying helps to preserve color.

#### 4.2.1. Applications

Compared to conventional drying techniques like sun drying, hot-air drying, etc., vacuum drying offers a number of advantages. It is more rapid, energy-efficient, and can help preserve the color, flavor, and aroma of the dried product. Vacuum drying also helps to preserve the volatile compounds present in medicinal plants, as it operates at lower temperatures compared to conventional drying methods. This method is highly beneficial for plants with delicate structures that can be easily damaged under high temperatures. Furthermore, the enzymatic breakdown and oxidation of bioactive components can be inhibited by vacuum drying [114]. But compared to other novel drying techniques, vacuum drying often demand longer drying periods and the usage of specialized equipment, such as vacuum pumps and chambers, which uses a lot of energy. The requirement of these specialized equipment also increases the initial capital investment making it expensive. The vacuum drying methods used today for medicinal plants are primarily based on practical experience rather than theoretical guidance, which results in poor drying quality and energy waste [82]. Consequently, it needs a certain amount of skill to be set up and operate efficiently.

### 4.3. Freeze Drying

**Figure 3:**
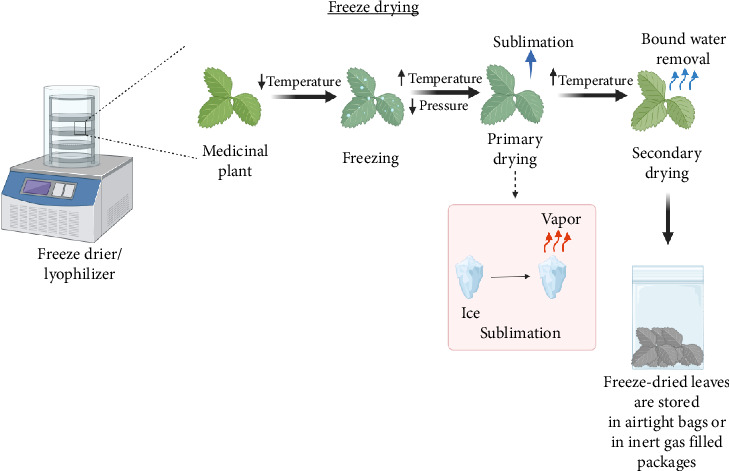
Schematic representation of freeze-drying process.

The process of freeze drying involves sublimating materials under vacuum to dry them. By operating at low temperatures, freeze-drying technology can decrease the loss of volatiles and provide a superior-quality dried plant material. There are two stages for freeze drying: primary drying and secondary drying [4]. Primary drying involves reducing pressure by vacuum and delivering heat to the substance for sublimating ice through conduction or radiation. Unfrozen water is removed during the secondary drying cycle [115]. The mechanism of freeze drying is explained in Figure 3. Compared to primary drying, the temperature will be increased in this stage to break any physicochemical associations that may have developed between the water molecules and the frozen matter. As it preserves the quality of the fresh material, freeze drying has recently become a crucial preservation technique for particularly delicate and heat-sensitive biological materials. Since low temperatures are used for drying, freeze drying results in minimal flavor and valuable component loss, and negligible shrinkage. Freeze drying is the best drying method for maintaining product quality since it uses a low processing temperature and less oxygen than other drying methods [116].

Research on the phytochemical properties of dried *Rosmarinus officinalis* L. suggests that freeze drying exhibited the best antidiabetic and antiaging activity among other drying methods [84]. A study on the impact of various drying techniques on the morphological structure, color profile and citral concentration of Lemongrass (*Cymbopogon citratus*) shows that a higher yield of powder was obtained by freeze-dried sample and freeze drying was able to retain the color of the lemongrass powder [76]. Additionally, freeze-dried lemongrass powder obtained had the highest concentration of citral compound. Freeze drying of stinging nettle leaves was found to have a higher amount of *β*-carotene and ascorbic acid content compared to the oven drying method [85]. A similar study on freeze drying of stinging nettle leaves also supports that high ascorbic acid is obtained by freeze drying compared to oven drying, but even though better phenolic compounds and antioxidant activity are obtained, it is still low compared to some other drying techniques [86]. Drying studies on saffron showed that freeze drying took the longest drying time, highest color values, maintained initial cellular structure and grain characteristics with the least amount of cell shrinkage, and retained the greatest amount of total crocins compared to other methods [80]. Compared to air or oven-drying, freeze drying has been demonstrated to better preserve camptothecin, a pyrroloquinoline alkaloid with antineoplastic effects, from *Camptotheca acuminata* [117].

As browning reactions are reduced in freeze drying, the color of the original sample can be maintained. Freeze drying increased the retention of anthocyanins, phenolic compounds, and antioxidant activity when compared to conventional drying techniques [118]. Despite being an excellent choice for preserving plant-based foods, freeze drying can result in a slight reduction in phytochemical content [119]. Through oxidative breakdown, freeze drying causes a reduction in lycopene content. If freeze drying is carried out in vacuum condition, this can be minimized. Since freeze drying process is done at a lower temperature, the bound lycopene which require mild heat treatment might not be released [120]. Even though this method is found to give highest amount of phenolic content, losses can occur during the freezing stage. This results from cell rupture (ice crystal formation) followed by the release of certain enzymes and activators that lead to breakdown of phenolic compounds [120]. Both aerobic and anaerobic processes can lead to the breakdown of vitamin C, while the latter normally happens at a slower rate. In freeze drying, low temperature is employed; therefore, temperature degradation is irrelevant; however, if vacuum is not applied, oxidative deterioration results in significant loss. Additionally, even if the second stage is conducted under vacuum, the freezing stage may still contain oxygen, which may favor these degradation reactions [121]. As a result of the autocatalytic oxidative reactions accelerated by the low water activities attained during freeze drying, lipid-based phytochemicals such as *β*-carotene, lycopene, vitamin E, and unsaturated oils can degrade, affecting the quality of the product. This is due to the possibility of free radical generation when there is environment devoid of liquid water and oxygen [119]. However, the phytochemical profile achieved by freeze drying is superior compared to all existing drying methods.

#### 4.3.1. Application

Freeze drying offers a gentle and effective preservation method by rapidly freezing the plants. In fact, during the freezing process, tiny ice crystals grow inside the cell and quickly expel themselves. Rapid ice crystal vaporization permits the preservation of cell structures and, as a result, the retention of phenolic compounds and vitamins, and complete evaporation of moisture for the long-term preservation of plant materials. Due to these qualities, freeze drying is frequently used as a pretreatment for plant materials [86]. By freeze drying, water is removed from the plant material, leaving behind a porous structure that enhances the extraction process. This freeze-dried plant material can be used as a starting point for the extraction of various bioactive compounds such as alkaloids, flavonoids, and terpenoids [122]. The porosity of the freeze-dried plants allows for increased solvent penetration, resulting in more efficient extraction. Additionally, the removal of water reduces the hydrolysis and oxidation reactions that may occur during the extraction process, thereby preserving the integrity and bioactivity of the extracted compounds. However, the extensive drying time, high cost, and energy consumption of freeze drying are major problems restricting its wider application [80]. The application of freeze drying at the industrial level is restricted to high-value products due to the higher cost of the process.

### 4.4. RW

**Figure 4:**
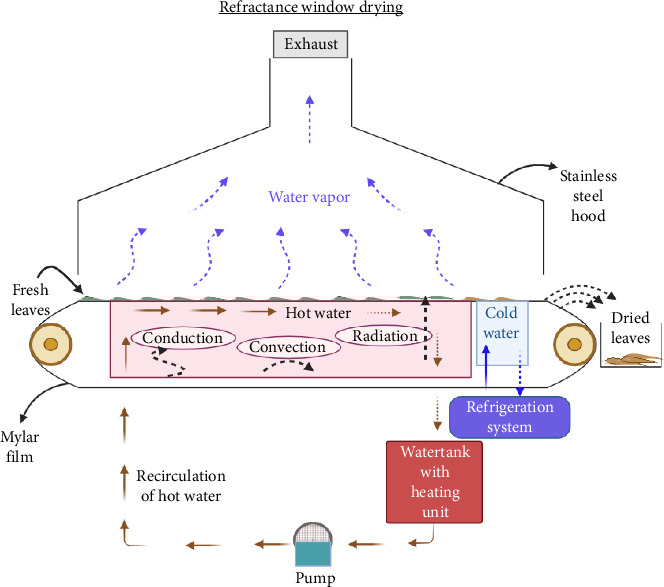
Pictorial representation of refractance window drying.

RW is a thin film drying technique and a rapidly developing drying process that facilitates drying through high heat and mass transfer rates [123]. The drying of food products takes place as a result of a combined mode of heat transfer such as conduction, radiation, and convection, making RW drying an energy-efficient (re-circulation of water) quick drying process [4]. During the RW drying process, Mylar film, an infrared transparent polyester plastic sheet, distributes radiative heat to food with a better drying rate, greater retention of nutrients, and minimal loss of aroma and flavor [124]. This film is placed in contact with water. The water molecules present in plant material act as windows and radiative heat transfer takes place directly through Mylar film. As the moisture content of plant material is considerably reduced, the window closes and further heat transfer is prevented. Therefore, there is no overheating of the material. The process of RW drying is explained in Figure 4. The dried product is produced with great quality since the temperature can be kept below 80°C, and degradation is prevented due to faster drying rate [123]. The use of RW dryers is no longer just restricted to the drying of food materials. It is now also being used in pharmaceutical, cosmetic, pigment, edible film development, and encapsulation. The phytochemical profile of plant material is influenced by drying factors such as drying duration, Mylar film thickness, and temperature [125].

RW drying employs a mild drying technique owing to its unique heating mechanism. Either we can use a low temperature like 60°C–70°C or a higher temperature of 90°C and above. The phytochemical composition of the dried product is significantly influenced by exposure time and temperature [126]. RW drying typically results in less change in color characteristics which is related to decreased pigment degradation, oxidation, phenolic polymerization, and enzymatic and nonenzymatic browning [127]. Lycopene, *β*-carotene, and ascorbic acid degradation occur more rapidly if we increase the drying temperature. Thermal deterioration and enzymatic oxidation are responsible for the decrease in ascorbic acid content [128]. In the same study, it was found that the main factors contributing to the decrease in lycopene concentration are lycopene isomerization and oxidation [128]. The presence of oxygen and high temperatures can cause carotenoid degradation. If enzymes such as polyphenol oxidase and lipoxygenase are not inactivated, they can result in oxidative changes causing degradation of *β*-carotene and other bioactive compounds [129].

Research on the potential to produce superior-quality aloe vera gel–dried slabs utilizing RW drying shows that significantly shorter drying times were obtained with higher quality retention in terms of color, vitamins C and E, and rehydration [87]. Another study on drying of *Dracocephalum kotschyi* using RW drying found to give higher total phenol content, TFC, antioxidant activity, and essential oil content [88]. Similar results were obtained during studies of RW drying of *Curcuma longa* (turmeric) [89], Malabar spinach [90], and Ginger [91].

#### 4.4.1. Application

Many culinary and medicinal herbs are traditionally dried to enhance flavor and prolong shelf life. However, the conventional methods of sun drying or air drying can lead to the loss of bioactive compounds and essential volatile substances, resulting in nutrient loss and flavor degradation. RW drying offers a solution to this problem by providing a gentle drying process that preserves the therapeutic properties, natural aroma, and flavor of the herbs. RW dryer limitations include its low throughput, the fact that it is primarily designed to dry liquids, the difficulty in handling powder with a high sugar content (stickiness), and its high cost [125]. Large surface areas are needed for drying and heat exchange during the dehydration of large amounts of material. Although the RW drying process is quick, the thickness of the film is kept on the low side, which lowers the process capacity [124].

### 4.5. Osmo Drying/Osmotic Dehydration

**Figure 5:**
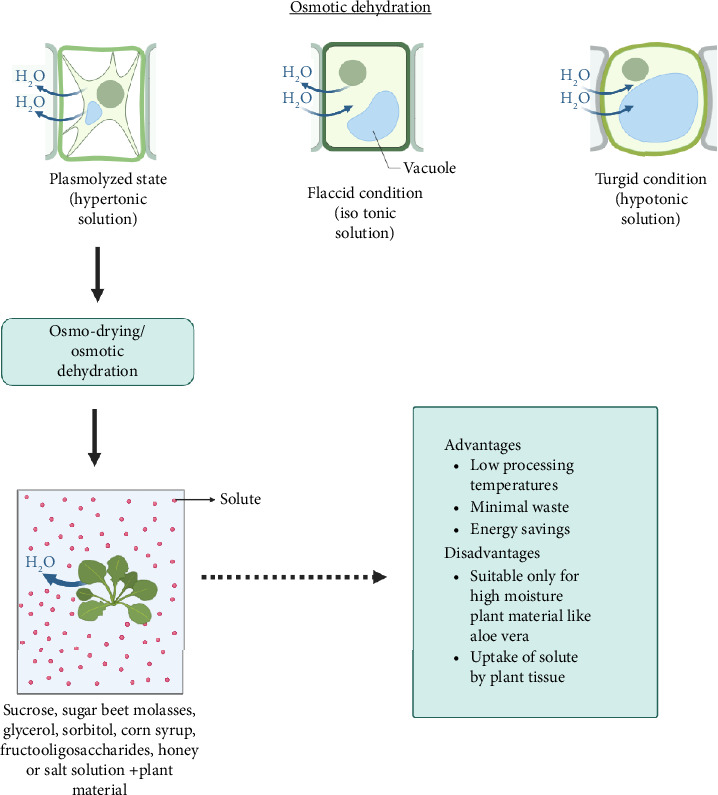
Graphical representation of osmotic dehydration process.

To increase the shelf life of fruits, vegetables, herbs, and other plant materials, osmotic dehydration is one of the suitable and efficient methods. Dehydration and an impregnation technique are combined in this method of drying. It is a procedure which involves removing a portion of the water from products to extend their shelf life [92]. This procedure works by immersing the product in a concentrated solution with a greater osmotic pressure, also known as a hypertonic solution, which simultaneously creates two strong counter-current flows [130]. The process of osmosis results in osmotic dehydration. The mechanism for water removal is the differential osmotic pressure between the plant tissue and the solution around it [131]. During this process, water diffuses from the plant tissue to the solution, while osmotic solute diffuses from the solution to the plant tissue. The process of osmo drying is explained in Figure 5.

Osmotic agent, solute concentration, agitation, temperature, soaking time, ratio of sample to solution, shape, size, and material compactness all have an impact on osmotic dehydration [130]. The most frequently used osmotic mediums are binary and ternary glucose or sucrose and salt solutions, sucrose, Sugar beet molasses, glycerol, sorbitol, corn syrup, fructo-oligosaccharides, honey, and maple syrup solutions [132]. Osmotic mediums should be carefully selected based on the type of material to be dehydrated. Osmotic treatment is a key approach for food dehydration that has a number of benefits, such as low processing temperatures, minimal waste, and energy savings. Oxygenated monoterpenes and oxygenated sesquiterpenes were mostly identified in the essential oils from samples that were subjected to osmotic treatment in a study on *Origanum vulgare* L. [92]. Osmotic dehydration of Aloe vera was found to reduce the initial moisture significantly depending on the concentration of the sugar solution used [93]. Similar results were obtained during the dehydration of ginger (*Zingiber officinale*) [94].

#### 4.5.1. Application

Osmotic dehydration is a nonthermal method that is mainly suitable for plant materials with high moisture content, like aloe vera. One of the significant applications of osmotic dehydration in medicinal plants is the preservation of their active compounds. Medicinal plants contain numerous bioactive compounds, such as alkaloids, phenolics, flavonoids, and essential oils, which have therapeutic properties. However, these compounds are often highly sensitive to external factors such as heat, light, and oxygen, which can lead to their degradation and loss of efficacy. Osmotic dehydration, by its nature, is a gentle preservation technique that can minimize the impact of these external factors on the active compounds of medicinal plants. Osmotic dehydration does have some disadvantages. The uptake of sugar will result in a reduction in acidity level, affecting the characteristics of some products, and also sugar coating is not desirable in the case of plant material. It has been discovered that osmotic dehydration is expensive when used in conjunction with other techniques such as vacuum drying, air drying, or microwave drying. Furthermore, the process is time-consuming [133]. Since it cannot achieve a desirable low water activity, this technique is used as a pretreatment method to reduce the initial moisture content of the plant material. Combining this osmotic dehydration with other conventional or novel drying method will improve the drying efficiency [134].

### 4.6. Supercritical CO_2_ Drying

**Figure 6:**
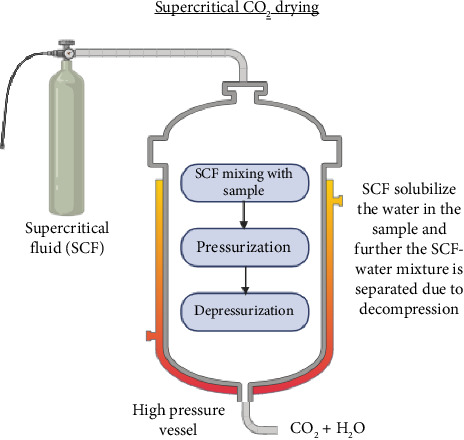
Schematic diagram of supercritical CO_2_ drying.

The unique properties of some substances above the critical point of temperature and pressure in phase equilibrium are used in supercritical fluid technology. They exhibit diffusivity similar to a gas and solvent power similar to a liquid. They are a perfect solvent for processing natural materials due to their physical properties. Supercritical carbon dioxide (scCO_2_), because it is mostly inert, affordable, nontoxic, nonflammable, high density, low viscosity, recyclable, available in high purity, leaves no residue, and is usually regarded as safe, is the most widely used supercritical fluid [135]. Additionally, CO_2_ has a low critical temperature, which can prevent product from thermal degradation. During the drying process, the supercritical CO_2_ is introduced into a sealed chamber containing the plant material. As the CO_2_ passes through the material, it dissolves the moisture present in the cells. Subsequently, the moisture-laden CO_2_ is released into a separate chamber, where the pressure is reduced, causing the CO_2_ to revert to its gaseous state. This results in the evaporation of the moisture, leaving behind dry plant material with preserved bioactive compounds. The mechanism of supercritical CO_2_ drying is explained in Figure 6. Supercritical carbon dioxide (scCO_2_) is a novel approach that is believed to guarantee both water removal and adequate microbial inactivation [95]. However, there is still not much evidence in the literature that the scCO_2_ drying method has an antibacterial effect. Microorganisms could be made inactive by scCO_2_ drying in a study on coriander leaves. The most susceptible microorganisms to the treatment were found to be yeast and molds, whereas mesophilic bacteria were significantly reduced [95]. The scCO_2_ extraction can be used to reduce the stress development in medicinal woods like eucalyptus [136]. Supercritical CO_2_ drying has been shown to have a positive impact on the stability and overall quality of bioactive compounds. By avoiding exposure to high temperatures and harsh solvents, this method prevents the degradation of sensitive compounds, such as volatile essential oils and delicate phytochemicals [137]. As a result, the medicinal plants retain their therapeutic properties, making them more valuable for pharmaceutical and nutraceutical applications. Additionally, supercritical CO_2_ drying minimizes oxidation, which can lead to the formation of harmful byproducts and the loss of antioxidant compounds [138]. The preservation of antioxidants in medicinal plants is crucial, as these compounds play a significant role in preventing cellular damage and promoting overall health. Supercritical CO_2_ drying ensures that the antioxidant capacity of the plants is maintained, enhancing their medicinal value [139]. But there is not much information regarding its effects on the physical and phytochemical properties of medicinal plants.

#### 4.6.1. Application

Supercritical CO_2_ drying is mainly used as a pretreatment to reduce moisture content of plant material with high moisture content such as aloe vera. The setup and operation of supercritical CO_2_ drying equipment can be costly. The specialized equipment required for this technique, including high-pressure vessels and extraction systems, can be expensive to acquire and maintain. Supercritical CO_2_ drying requires precise control of temperature and pressure conditions. Scaling up supercritical CO_2_ drying processes for large-scale production can be challenging. The equipment and infrastructure required to handle larger volumes of plant material can be challenging and expensive. This limitation may restrict the application of supercritical CO_2_ drying to smaller batches or specialized products [140].

### 4.7. Spray Drying

**Figure 7:**
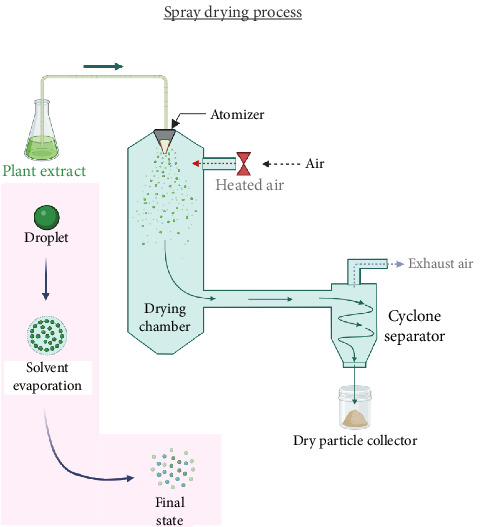
Schematic diagram of spray drying process.

A well-known, efficient, in terms of energy, and scalable drying method is spray drying, which converts liquids directly into powder [141]. An extract of medicinal plant material as a fluid stream is sprayed and dried to produce solid particles during spray drying. It is a special process called atomization that disperses the fluid at faster evaporation rates based on the formation of fine sprays to speed up the drying process [142]. Another benefit is the ability to produce a free-flowing powder with a limited particle size distribution by carefully managing the operational settings [143]. The process of spray drying is diagrammatically expressed in Figure 7. When compared to other traditional drying methods, the spray drying technique typically results in a significant reduction in drying time. The design of the equipment, process parameters, and fluid feed characteristics can all have a significant impact on the qualities of the spray-dried powder [144].

The spray-dried extract has been found to have superior antioxidant, anti-inflammatory, and antidiabetic activities in a comparative study. Because it is easy, faster to produce, and generally has cheaper production costs than freeze drying, it was also discovered to be a superior option than freeze drying [145]. The water activity of spray-dried Moringa leaf powder was found to be less than 0.6, which is below the threshold for spoiling [97]. Additionally, when it comes to phytochemical retention, spray drying increased antioxidant activity, TPC, and total chlorophyll content. Similar research on spray-dried extract of Moldavian balm showed that rising inlet air temperature resulted in lower moisture content, total phenol content, TFC, and antioxidant activity [96].

#### 4.7.1. Application

Spray drying is a widely used technique in the pharmaceutical industry for the production of various medicinal products from natural sources. This process involves the conversion of liquid extracts obtained from medicinal plants into dry powder form, which can be easily stored, transported, and formulated into different dosage forms [146]. The spray drying method has several advantages over other drying techniques, as it allows for the preservation of the active compounds in the plant extracts, improves their stability, and enhances their bioavailability. Some of the common challenges with spray drying include the complexity of the equipment, loss of volatile components, high equipment and operational costs, and difficulty in atomization of high-viscosity liquids [147]. As a successful approach for emulsifying and extracting bioactive compounds from plant material, ultrasound can be used as a pretreatment step prior to spray drying [143].

### 4.8. Hybrid Drying Techniques

To meet consumer demands, but also government energy policies, higher production throughput, better process economics, and environmental sustainability guidelines, more than one drying mechanism is integrated into a single operation (hybrid arrangement) [148]. By optimizing the effectiveness of each drying technique, hybrid dryers are able to produce dried products with exceptional quality attributes like color, texture, flavor, aroma, and rehydration capacity. They also preserve the nutritional and bioactive compounds.

Hot-air-microwave drying is a two-step drying method that involves initial hot-air drying, followed by intermittent microwave drying [149]. Hot-air drying is widely employed due to its simplicity and cost-effectiveness. However, it is a relatively slow process and may lead to undesired degradation of bioactive compounds and volatile essential oils present in medicinal plants. On the other hand, microwave drying is known for its rapid and uniform drying capabilities [150]. By combining these two methods, the drying time of medicinal plants can be significantly reduced while ensuring minimal loss of bioactive compounds. The hot air provides the initial drying surface, enabling faster moisture removal, and the microwaves penetrate deep within the plant material, further accelerating the drying process [151]. The initial reduction in moisture saves energy consumption and improves the quality of the plant material. A study on the effect of various drying methods revealed that microwave-assisted hot-air hybrid drying was preferred for *Cymbopogon citratus* and *Psophocarpus tetragonolobus* [75].

Another widely studied hybrid drying method is the vacuum-microwave drying. Vacuum-microwave drying combines vacuum drying with microwave heating. This combination is thought to be suitable for the drying process to take place in a low-pressure environment, hence reducing oxidation processes by lowering the boiling point of water [152]. It has been studied and found highly efficient in drying of many medicinal plants such as rosemary (*Rosmarinus officinalis* L.) [84], *Cassia alata* [153], *Strobilanthes crispus* [152], *Moringa oleifera* [154], etc. Advantages of microwave vacuum drying include a faster drying rate, less heating in regions with less water, resulting in reduced overheating and preventing significant quality degradation [155]. At the same time, microwave-vacuum drying is expensive due to the requirement of vacuum installation and nonuniform temperature distribution may occur while drying thick plant materials.

Another hybrid drying technique involves the integration of freeze drying and vacuum drying. Freeze drying is widely regarded as one of the best methods for preserving the bioactivity of medicinal plants. It involves freezing the plant material followed by sublimation of ice crystals under vacuum conditions. This process removes moisture without significant heat exposure, thus minimizing the loss of bioactive compounds [156]. However, freeze drying is a lengthy and energy-intensive process. By combining vacuum drying with freeze drying, the drying time can be reduced while still maintaining the advantages of low-temperature drying. The vacuum drying step helps in eliminating the remaining moisture content efficiently, enhancing the overall drying efficiency. But due to high capital investment, its use in drying medicinal plants is limited. This method is mainly used for drying high-value fruits [119].

#### 4.8.1. Application

The hybrid drying techniques have various applications in the drying of medicinal plants. One significant application is the drying of delicate and heat-sensitive plants. Traditional drying methods, such as sun drying or oven drying, may cause excessive heat exposure, resulting in the degradation of bioactive compounds [7]. By combining low-temperature techniques, such as freeze drying or microwave drying, with conventional drying methods, the desired results can be achieved without compromising the quality of the medicinal plants [75]. But combining drying methods will definitely increase the cost.

Therefore, the development and application of innovative drying methods have greatly expanded the possibilities for drying medicinal plants. Techniques such as microwave drying, vacuum drying, freeze drying, osmotic dehydration, RW drying, and supercritical CO_2_ drying each offer distinct advantages and limitations. Microwave drying is known for its rapid and efficient drying capabilities making it ideal for bulk drying and preserving the bioactive compounds found in medicinal plants. Vacuum drying operates under reduced pressure to minimize heat exposure and maintain the integrity of heat-sensitive compounds. Freeze drying retains the structure, flavor, and bioactive constituents of medicinal plants by removing moisture through sublimation. Osmotic dehydration uses osmotic solutions to selectively extract moisture from medicinal plants while still preserving their quality and properties. RW drying uses infrared radiation for gentle and high-quality preservation of color, flavor, and nutrients in medicinal plants. Supercritical CO_2_ drying utilizes carbon dioxide in its supercritical state to offer gentle conditions for effective drying without the need for solvents. Hybrid drying techniques offer potential solutions by overcoming drawbacks and combining the strengths of multiple drying methods. Each of the mentioned method has its advantages and limitations that should be considered while choosing a suitable technique. Factors such as the characteristics of the medicinal plants, desired product quality, processing capacity, and economic feasibility all play a role in determining the most appropriate method. Continued research, optimization, and innovation in drying technologies hold great promise for further advancements in the field. These advancements will undoubtedly contribute to not only increased availability but also improved efficacy of medicinal plant–based herbal medicines and natural products.

## 5. Conclusion

The moisture content of aromatic and medicinal plants has a major impact on their physical and chemical properties. It determines the stability and quality of these plants. Therefore, drying, or the removal of water, is the first stage in many postharvest operations [157]. It is difficult to choose a consistent drying technique for all the medicinal plants, because each plant has distinct biostructures and volatile chemicals. These characteristics may have an impact on the way these plants react to various drying methods, which may have an impact on the extent to which their bioactive components are preserved. Consequently, a specific drying technique is necessary to preserve the quality and effectiveness of each medicinal plant [158].

Compared to conventional techniques, microwave drying has several advantages such as shorter drying time, better retention of bioactive components, color, and aroma. However, its limitations include potential non-uniform heating, which can lead to uneven drying and degradation of sensitive compounds, especially at higher power levels or prolonged exposure [102]. Vacuum drying of medicinal plants has been shown to yield extracts with high polyphenol and flavonoid content, excellent color retention, and superior rehydration capacity [78]. Freeze drying is a superior method for preserving the bioactive compounds, color, and aroma of medicinal plants, offering advantages such as extended shelf life, minimal nutrient degradation, and enhanced rehydration properties. High operating costs, extended processing time, and the possibility of particular compounds, such as phenolics and carotenoids, degrading as a result of oxidative or enzymatic activity throughout the process are some of its drawbacks [120]. RW drying typically results in less change in characteristics, which is related to decreased pigment degradation, oxidation, phenolic polymerization, and enzymatic and nonenzymatic browning [127]. Osmotic dehydration is a nonthermal method that is mainly suitable for plant materials with high moisture content, and hence it preserves the temperature-sensitive bioactive compounds [133]. By avoiding exposure to high temperatures and harsh solvents, supercritical CO_2_ drying prevents the degradation of sensitive compounds, such as volatile essential oils and delicate phytochemicals [137]. When it comes to phytochemical retention, spray drying increased antioxidant activity, TPC, and total chlorophyll content [96]. To enhance drying efficiency and preserve the quality of medicinal plants, hybrid drying techniques combine two or more methods, such as microwave-vacuum, vacuum-freeze, etc.

There is a substantial gap between academic research and its practical industrial application, even in highly developed countries where independent education and research funds are widely available. Especially on an industrial scale, this conflict frequently leads to the underutilization of important research findings. As a result, this discrepancy between the results of academic research and industrial application hinders both economic development and the advancement of technology [158]. The full potential of research can be achieved through improving communication, coordinating goals, allocating resources, and encouraging teamwork, which will result in innovation and economic growth.

## Figures and Tables

**Table 1:** Common medicinal plants, their bioactive compounds, and important pharmacological effects.

Sl no	Medicinal plants	Bioactive compound	Therapeutic effects	References
1	*Ocimum sanctum* (tulsi)	Carvacrol, eugenol, methyl eugenol, caryophyllene	Antibacterial, antimalarial	[16]
		Ursolic acid, oleanlic acid	Anticancer, adaptogen	
		Rosmarinic acid, pegenin, eugenol	Antioxidant, anti-inflammatory	[17]

2	Aloe vera (*Aloe barbadensis miller*)	Aloin	Anti-inflammatory, skin protection, bone protection	[18]
		Aloe-emodin	Anticancer, antidiabetic, bone protection, antimicrobial	
		Homonataloin, aloinoside, microdontin	Laxative, antidiabetic, antioxidant	[19]

3	Ginger (*Zingiber officinale*)	Gingerol	Anti-inflammatory, antioxidant, anticancer	[20]
		Shogaols, paradols	Anti-inflammatory, anticancer	[21]
		Zingerone	Antimicrobial	

4	Turmeric (*Curcuma longa*)	Curcumin, demethoxycurcumin, bisdemethoxycurcumin	Anti-inflammatory, antioxidant, anticancer	[22]
		Turmerones	Anti-inflammatory, anticancer	
		Atlatone, germacrone	Anti-inflammatory	[23]
		Zingiberene, curcumenol	Anti-inflammatory, antimicrobial	

5	Gotu kola (*Centella asiatica*)	Asiaticoside	Anti-inflammatory, antioxidant, neuroprotective	[24]
		Madecassoside, madecassic acid	Anti-inflammatory, antioxidant, antirheumatic, wound healing	[25]
		Asiatic acid	Anti-inflammatory, antioxidant, anticancer, antihypertensive, cardioprotective	
		Centelloside, centroside, thankuniside, isothankuniside, brahmoside	Anti-inflammatory, antioxidant	[26]
		Bacoside A	Neuroprotective property, antioxidant	[27]

6	Ginkgo (*Ginkgo biloba*)	Ginkgolides, bilobalide	Anti-inflammatory, antioxidant, neuroprotective	[28]
		Quercetin, kaempferol, isorhamnetin, amentoflavone	Antioxidant, anticancer, anti-inflammatory	[29]
		Ginkgetin, proanthocyanidins, catechins	Antioxidant, anticancer	[30] [31]

7	Peppermint (*Mentha piperita*)	Hesperidin, luteolin, caffeic acid	Antioxidant, anticancer, anti-inflammatory	[32]
		Menthol	Antimicrobial, anti-inflammatory, analgesic	
		Rosmarinic acid	Antioxidant, anti-inflammatory, neuroprotective	
		Eriocitrin	Antioxidant, anti-inflammatory	
		Menthone, isomenthone	Antimicrobial	
		Pulegone, menthyl acetate	Antimicrobial, insecticidal	

8	Lemon grass (*Cymbopogon ambiguus*)	Citral, linalool	Antimicrobial, antioxidant, anti-inflammatory, analgesic, sedative, anticancer	[33]
		Myrcene	Analgesic, anti-inflammatory	
		Geraniol, nerol, neral	Antimicrobial, antioxidant	[34]
		*β*-caryophyllene, *α*-pinene	Anti-inflammatory	
		Citronellal	Antimicrobial	

9	Sage (*Salvia officinalis*)	Carnosic acid	Antioxidant, anti-inflammatory, neuroprotective	[35]
		Carnosol, ursolic acid, apigenin, luteolin, quercetin, kaempferol	Antioxidant, anti-inflammatory, anticancer	[36]
		Rosmarinic acid	Anti-inflammatory, antioxidant, antimicrobial	[37]
		Salvianolic acid B, tanshinone IIA	Antioxidant, anti-inflammatory	[38]

10	Thyme (*Thymus vulgaris*)	Thymol, carvacrol	Antimicrobial, antioxidant, anti-inflammatory	[39]
		Ursolic acid, apigenin, luteolin, quercetin, kaempferol	Antioxidant, anti-inflammatory, anticancer	[40]
		Caffeic acid, tannins	Antioxidant, anti-inflammatory	[41]

11	Lavender (*Lavandula angustifolia*)	Linalool, linalyl acetate	Anti-inflammatory, antioxidant, sedative properties	[42]
		Limonene	Anti-inflammatory, antioxidant, anticancer	[43]
		Perillyl alcohol	Anticancer, anti-inflammatory	
		Cis-smine, caffeic acid	Antioxidant, anti-inflammatory	

12	Fenugreek (*Trigonella foenum-graecum*)	Galactomannan	Antidiabetic, antioxidant, immunomodulatory properties	[44]
		4-hydroxyisoleucine	Antidiabetic, hypolipidemic properties	

13	Indian barberry (*Berberis aristata*)	Berberine	Antimicrobial, anticancer, antidiabetic, anti-inflammatory, anti-oxidant, anti-tumorigenic, neuroprotective, antidiarrheal	[45]
		Jatrorrhizine	Antidiabetic, antimicrobial, anti-obesity, anti-protozoal, anticancer, hypolipidemic	[46]
		Oxyacanthine	Anticancer, antidiabetic, anti-inflammatory, antimicrobial, antioxidant	
		Palmatine	Anti-cancer, antidiabetic, anti-inflammatory, antimicrobial, antioxidant, hepatoprotective	

14	Bengal quince (*Aegle marmelos*)	Coumarins (marmin, marmesin, marmesinin)	Antioxidant, anticancer, gastroprotective, antidiarrheal, cardioprotective, antifertility	[47]
		Lupeol	Antiproliferative, anticancer, chemopreventive, anti-inflammatory	[48]
		Eugenol	Antiproliferative, anticancer, chemopreventive, antioxidant, hepatoprotective	
		Citral	Antiproliferative, anticancer, chemopreventive	
		Marmin (alkaloid), fagarine	Antifertility	
		Skimmianine	Anti-inflammatory, antimalarial, analgesic, antipyretic	

15	Ashwagandha (*Withania somnifera*)	Withaferin-A	Neuroprotective, cardioprotective, anti-inflammatory, anti-Parkinson, anticancer, anti-Alzheimer, hepatoprotective	[49] [50]
		Withanolide-A	Neuroprotective, antiepileptic, antioxidant, anticancer, immunomodulatory	[51]
		Withanolide-D, E, F	Anticancer	
		Withanone	Anti-inflammatory	
		Withanoside-V, X	Antiviral	

**Table 2:** Novel drying methods, drying conditions, and its use in medicinal plant.

Sl. no	Drying technique	Conditions	Effects	Plants	References
1	Microwave drying	500–1200 W/1.5–3 h	Increased antioxidant activity, total phenolic content, total flavonoid content, retains aroma components	Galangal (*Alpinia galanga* L.)	[10]
				Aloe vera (*Aloe barbadensis Mill*)	[75]
				*Centella asiatica* (L.) urban	[75]
				*Cymbopogon citratus* (DC.)—lemongrass	[76]
				*Psophocarpus tetragonolobus* (L.)- winged bean	[75]
				*Matricaria chamomilla*-Chamomile	[77]

2	Vacuum drying	40–60°C/5–24 h/15 kPa	Reduced degradation of ascorbic acid, lycopene, and pigments Decreased browning reactions Increased antioxidant activity, total phenolic content, total flavonoid content	Peppermint (*Mentha piperita* L.)	[78]
				Sweet basil (*Ocimum basilicum* L.)	[78]
				Lemon balm (*Melissa officinalis* L.)	[78]
				Lemongrass (*Cymbopogon citratus*)	[76]
				Sabah snake grass (*Clinacanthus nutans*)	[79]
				Saffron (*Crocus sativus* L.)	[80]
				*Anoectochilus roxburghii* (Wall.)	[81]
				*Callicarpa nudiflora*	[82]

3	Freeze drying	65–200 Pa, −20 to −60°C	Reduced degradation of ascorbic acid, and pigments Decreased browning reactions Increased anthocyanins, phenolic compounds, and antioxidant activity	Aloe vera (*Aloe barbadensis Mill*)	[75]
				*Centella asiatica* (L.) urban	[75]
				Lemongrass (*Cymbopogon citratus*)	[76]
				Eucalyptus (*Eucalyptus globulus*)	[83]
				Rosemary (*Rosmarinus officinalis*)	[84]
				Stinging nettle leaves (*Urtica dioica* L.)	[85]
				Saffron (*Crocus* sativus L.)	[86]
				*Anoectochilus roxburghii* (Wall.)	[80]

4	Refractance window drying	125–350 μm (Mylar film thickness) 60°C–90°C	Improved retention of lypopene and ascorbic acid Increased total phenol content, total flavonoid content, and antioxidant activity	Aloe vera (*Aloe barbadensis Mill*)	[87]
				*Dracocephalum kotschyi*	[88]
				Turmeric (*Curcuma longa*)	[89]
				Malabar spinach (*Basella alba*)	[90]
				Ginger (*Zingiber officinale*)	[91]

5	Osmotic dehydration	Sample-to-solution ratio-1:20 (w/w) 20°C–40°C	Retains heat-sensitive bioactive compounds	Oregano (*Origanum vulgare* L.) Aloe vera (*Aloe barbadensis Mill*) ginger (*Zingiber officinale*)	[92] [93] [94]

6	Supercritical CO_2_ drying		More studies are required	Eucalyptus	[53]
				Coriander (*Coriandrum sativum*)	[95]

7	Spray drying	Inlet temperature 120°C–190°C	Retains pigments	Moldavian balm (*Dracocephalum moldavica* L.)	[96]
		Outlet temperature 50°C–80°C	Increased total phenol content, total flavonoid content, and antioxidant activity	Moringa (*Moringa oleifera* Lam)	[97]

## Data Availability

Data sharing is not applicable to this article as no new data were created or analyzed in this study.
